# Combined sodium ion sensitivity in agonist binding and internalization of vasopressin V1b receptors

**DOI:** 10.1038/srep25327

**Published:** 2016-05-03

**Authors:** Taka-aki Koshimizu, Aki Kashiwazaki, Junichi Taniguchi

**Affiliations:** 1 Division of Molecular Pharmacology, Department of Pharmacology, Jichi Medical University, Tochigi 329-0498, Japan

## Abstract

Reducing Na^+^ in the extracellular environment may lead to two beneficial effects for increasing agonist binding to cell surface G-protein coupled receptors (GPCRs): reduction of Na^+^-mediated binding block and reduce of receptor internalization. However, such combined effects have not been explored. We used Chinese Hamster Ovary cells expressing vasopressin V1b receptors as a model to explore Na^+^ sensitivity in agonist binding and receptor internalization. Under basal conditions, a large fraction of V1b receptors is located intracellularly, and a small fraction is in the plasma membrane. Decreases in external Na^+^ increased cell surface [^3^H]AVP binding and decreased receptor internalization. Substitution of Na^+^ by Cs^+^ or NH_4_^+^ inhibited agonist binding. To suppress receptor internalization, the concentration of NaCl, but not of CsCl, had to be less than 50 mM, due to the high sensitivity of the internalization machinery to Na^+^ over Cs^+^. Iso-osmotic supplementation of glucose or NH_4_Cl maintained internalization of the V1b receptor, even in a low-NaCl environment. Moreover, iodide ions, which acted as a counter anion, inhibited V1b agonist binding. In summary, we found external ionic conditions that could increase the presence of high-affinity state receptors at the cell surface with minimum internalization during agonist stimulations.

G protein-coupled receptors (GPCRs) receive a broad range of information, mediated by diverse agonists binding at the active (orthosteric) sites of the receptor. The affinity of orthosteric agonist binding to GPCRs can be allosterically modulated by the ionic composition of extracellular fluids^1^. Na^+^ allosterically inhibits the binding of agonists, but not antagonists, in many class A GPCRs^2^,^3^,^4^. The structural basis of the interactions between GPCRs and Na^+^ has been demonstrated by the solution structure of the human A_2a_ adenosine receptor at 1.8 angstrom high resolution^5^. Na^+^ and the surrounding water molecule network interact with well-preserved amino acids in the Na^+^-binding pocket, which is created by receptor transmembrane helixes. The introduction of amino acid substitutions to a conserved aspartic acid [Asp^2.50^ in Ballesteros-Weinstein numbering^6^] in the second transmembrane domain of neurotensin receptors abrogates the Na^+^ effect on ligand binding^3^,^7^. A comparison between active and inactive A_2a_ receptors has indicated that high-affinity agonist binding disrupts the interaction between the receptor and the Na^+^-water network^5^.

In addition to the modulation of agonist binding by Na^+^, receptor internalization can be regulated by extracellular tonicity and ion conditions. Hypotonic shock and the subsequent reduction in extracellular potassium ion levels effectively depletes intracellular potassium ions and blocks internalization of the receptor for low density lipoprotein (LDL) and epidermal growth factors (EGF), which may be a result of the inhibition of new clathrin pit formation^8^,^9^. The molecular mechanism and structural requirements for sensing intracellular potassium in clathrin-coated pit formation are not well-understood. For plasma membrane GPCRs, an activated receptor interacts with the corresponding heterotrimeric G proteins^10^. The agonist-bound receptors are subsequently phosphorylated, associate with β-arrestin proteins and are internalized by clathrin-dependent and independent mechanisms^11^. Hyperosmotic conditions produced by high glucose have also been reported to inhibit the internalization of GPCRs and other receptors^12^. Because Na^+^ is one of the major determinants of extracellular fluid osmolarity, changing extracellular Na^+^ levels may alter both the agonist binding and internalization processes in GPCRs. However, the overall effect of changing the levels of extracellular Na^+^ ions on the internalization of GPCRs in a cellular context has not been examined in detail.

Using the vasopressin V1b receptor expressed in Chinese hamster ovary (CHO) cells as a model, we quantitatively investigated the interaction between the agonist and cell surface receptors and subsequent receptor internalization in a modified extracellular ionic environment composition. Specifically, the effects of decreasing the extracellular Na^+^ level, replacing Na^+^ with other monovalent cations, or replacing Na^+^ with isotonic glucose on both the agonist binding and internalization of the V1b receptors were examined. The posterior pituitary hormone arginine-vasopressin (AVP) is a nonapeptide that strongly promotes water retention in normal and pathological conditions^13^,^14^. Neurohormonal stimuli of AVP are perceived by three vasopressin receptor subtypes (V1a, V1b and V2) as well as the oxytocin receptor^15^,^16^. V1b receptor expression is found in peripheral endocrine organs, such as the anterior pituitary, pancreatic β-cells, and the adrenal medulla. In the central nervous system, the V1b receptor is also expressed in the hippocampus, the paraventricular nucleus and the olfactory bulb regions^17^. In addition, the V1b receptor is located in the colonic epithelia, where the ionic composition around the cells can be widely altered in physiological and pathophysiological conditions^18^,^19^.

Here, we measured [^3^H]AVP uptake into CHO cells expressing the V1b receptors to evaluate V1b receptor internalization, on the basis of our recent findings that the V1b receptors are distributed in both the cytoplasm and the plasma membrane at resting state and that they extensively internalized agonist when stimulated^20^. We evaluated agonist binding and internalization under a wide range of external Na^+^ levels and identified an unexpected robustness of the receptor function in a cellular context.

## Results

### Agonist binding and internalization of V1b receptors were dependent on extracellular Na^+^ levels

**Figure 1:**
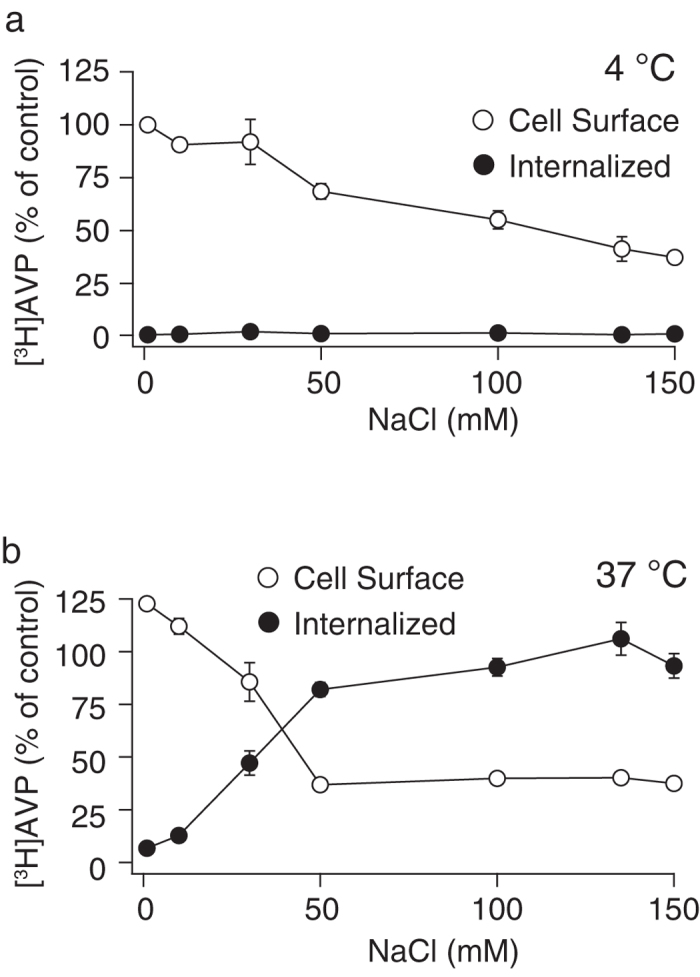
Sodium chloride dependency of [^3^H]AVP binding and internalization in CHO/V1b cells. Cells that stably expressed V1b receptors were incubated with 1 nM [^3^H]AVP in different NaCl concentrations at 4 °C for 2 hours to establish the binding equilibrium. The cells were further incubated at 4 (**a**) or 37 °C (**b**) for 30 min in the presence of various concentrations of external NaCl and were washed with buffer to remove the unbound ligands. No other solutes were added when the external NaCl concentration was decreased. Washing the cells with acidic washing buffer removed the [^3^H]AVP bound to the cell surface receptors, and the acidic buffer was mixed with scintillation cocktail to measure radioactivity (open circle). The cells were lysed after an acid wash and the remaining counts associated with the cells were measured (filled circle). For the detection of non-specific binding, 1 μM AVP was included in the binding buffer. The data are expressed as the percentage of the cell surface counts obtained from the cells incubated in buffer with no added NaCl at 4 °C. The data were obtained from five to seven independent experiments.

Agonist binding and receptor internalization by clathrin-coated pits are two important steps dependent on the extracellular NaCl concentration^3^,^21^. Using [^3^H]AVP, agonist binding was evaluated by the radioactivity of [^3^H]AVP bound to cell surface receptors, whereas receptor internalization was evaluated by the radioactivity of [^3^H]AVP taken up by CHO cells^20^. We initially examined the NaCl dependency of agonist binding to the V1b receptor at 4 °C, in which the V1b receptor internalization was inhibited. The binding equilibrium was established by incubating the CHO/V1b cells with 1 nM [^3^H]AVP in different concentrations of NaCl for 2 hours at 4 °C. An increase in the NaCl concentrations from 0 to 150 mM in the buffer resulted in a decrease in the specific [^3^H]AVP binding to 37% of the counts measured in 0 mM NaCl (Fig. 1a). Therefore, the V1b receptor was considered to be a suitable model to examine Na^+^ sensitivity in agonist binding. Washing the cells with acidic buffer effectively removed more than 99% of the counts bound to the cells, when the cells were incubated at 4 °C. This finding indicates that the majority of the radioligands were bound to the cell surface V1b receptor and sensitive to acid washing^20^.

The cells incubated with [^3^H]AVP for 2 hours at 4 °C were transferred to 37 °C to examine receptor internalization in the buffer conditions with different concentrations of NaCl. After incubation at 37 °C for 30 min, the radioactivity of [^3^H]AVP either at the cell surface or in the cytoplasm of CHO/V1b cells was measured. The values were calculated as a percentage relative to the counts of [^3^H]AVP obtained in the cells incubated at 4 °C with no external NaCl. At the physiological concentration of 135 mM NaCl, a large fraction of the counts (106 ± 8%, n = 5) were from acid-resistant, internalized [^3^H]AVP (Fig. 1b). Therefore, the V1b receptors undergo extensive internalization during incubation at 37 °C. Because of Na^+^-dependent blocking of agonist binding, we initially expected that the agonist internalization would be gradually inhibited by increasing the Na^+^ levels in the buffer. However, we obtained the opposite result: extensive internalization of the V1b receptor was detected in a wide range of NaCl between 50–150 mM (Fig. 1b). When NaCl was below 50 mM, the internalized radioligands sharply decreased and the cell surface ligands increased (Fig. 1b). In terms of the efficiency of signal transduction from activated receptors, NaCl in the external buffer was necessary for IP_3_ production. In the NaCl-containing buffer, the IP_3_ level was increased by 100 nM AVP from 6.6 ± 1.2 nM at basal to 13.2 ± 0.7 nM (n = 3). In contrast, in the buffer without NaCl, both the basal and AVP-stimulated IP_3_ levels were below the detection level (<1 nM).

### Sodium ion was preferred compared with cesium ion for the internalization of V1b receptors

**Figure 2:**
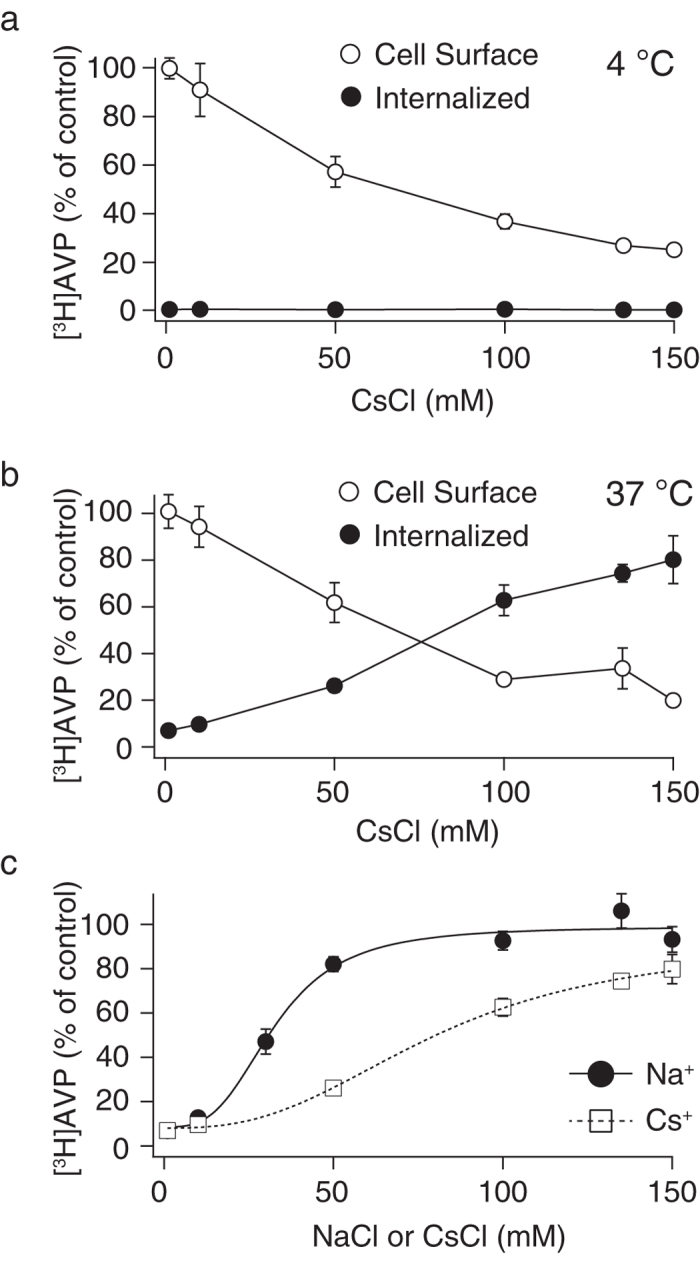
Cesium chloride dependency of [^3^H]AVP binding and internalization in CHO/V1b cells. CsCl was used instead of NaCl in the experiments shown in Fig. 1. CHO/V1b cells were incubated with 1 nM [^3^H]AVP in buffer with different CsCl concentrations at 4 °C for 2 hours to establish the binding equilibrium. The cells were further incubated at 4 (**a**) or 37 °C (**b**) for 30 min and washed with neutral buffer to remove the unbound ligands. Washing the cells with acidic buffer removed the [^3^H]AVP bound to the cell surface receptor, and the acidic buffer was mixed with scintillation cocktail for measurement of radioactivity (open circle). The cells were lysed after an acid wash, and the remaining counts associated with the cells were measured (filled circle). For the detection of the non-specific binding, 1 μM AVP was included in the binding buffer. The data are expressed as the percentage of cell surface counts obtained from the cells incubated in buffer with no added CsCl at 4 °C. The data were obtained from three independent experiments. In Fig. 2c, the data points were obtained from the internalized [^3^H]AVP in the NaCl- or CsCl-containing buffer shown in Fig. 1b or 2b and were fitted to Hill’s four parameter logistic equation using the computer program Igor. The number of the binding sites of Na^+^ and Cs^+^ during the V1b receptor internalization was assumed as one site.

We substituted Na^+^ for Cs^+^ in the external buffer and examined the AVP binding and internalization in CHO/V1b cells. As shown in Fig. 2a, increasing the CsCl concentration in the binding buffer at 4 °C resulted in the progressive inhibition of [^3^H]AVP binding, as demonstrated in the NaCl buffer (Figs 1a and 2a). This result suggests that extracellular Na^+^ can be substituted with Cs^+^ to inhibit [^3^H]AVP binding to the V1b receptors. However, in contrast to the stable distribution of the cell surface and internalized ligand in various NaCl concentrations from 50 to 150 mM (Fig. 1b), an increase in the CsCl concentration from 0 to 150 mM in the external buffer resulted in a gradual increase in the [^3^H]AVP internalization and a contrasting decrease in the cell surface binding (Fig. 2b), whereas the [^3^H]AVP internalization was almost fully enhanced by increasing the NaCl concentration from 0 to 50 mM. Therefore, Na^+^ was more effective than Cs^+^ in promoting receptor internalization (Fig. 2c). Data points for internalized [^3^H]AVP in the different binding buffers were fitted to Hill’s equation, and the NaCl or CsCl concentrations that caused half the maximum internalization were calculated (31 ± 3 and 81 ± 7 mM for NaCl and CsCl, respectively, Fig. 2c). Furthermore, Hill’s co-efficient values (n_H_) were 3.2 and 2.7 for the NaCl and CsCl buffer, respectively (Fig. 2c), indicating that cooperativity of Na^+^ is higher than Cs^+^ in promoting receptor internalization.

**Figure 3:**
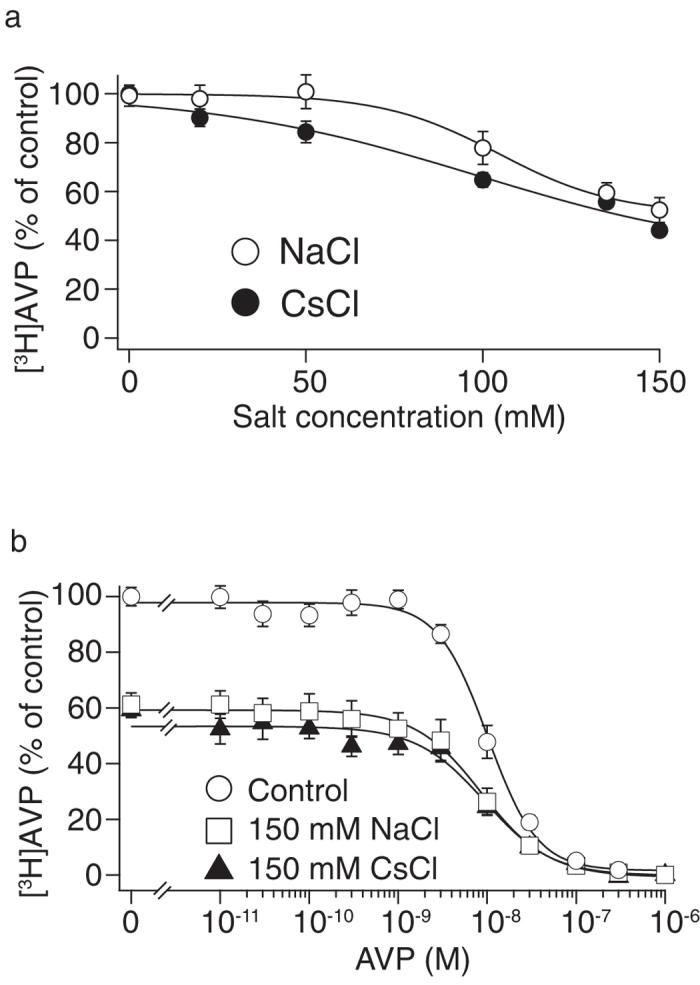
NaCl and CsCl inhibited [^3^H]AVP binding to membrane samples from CHP/V1b cells. (**a**) The samples prepared from plasma membrane (70 μg) and 1 nM [^3^H]AVP were incubated at 25 °C in a volume of 400 μL of binding buffer containing various concentrations of NaCl or CsCl. The data are expressed as the percentage of specific binding obtained in the buffer with no added NaCl or CsCl. (**b**) The effects of NaCl and CsCl were examined in competition binding experiments using 1 nM [^3^H]AVP and various concentrations of cold AVP. Membrane samples (70 μg), [^3^H]AVP and cold AVP were incubated in 300 μL of buffer B (Control) or buffer B containing 150 mM NaCl (NaCl) or CsCl (CsCl). The data are expressed as the percentage of specific binding obtained in the buffer B with no added NaCl, CsCl or cold AVP. Nonspecific binding was determined in the presence of 1 μM AVP. The points in the graph represent the results from four experiments performed in duplicate.

In the [^3^H]AVP binding study using membrane samples from CHO/V1b cells, we examined the direct effect of changing NaCl or CsCl levels on the interaction between agonist and V1b receptors. As shown in Fig. 3a, 150 mM CsCl inhibited [^3^H]AVP binding to a similar level as 150 mM NaCl. [^3^H]AVP binding was inhibited by 150 mM of NaCl or CsCl to 44 ± 1 or 52 ± 5 %, respectively (n = 8). In the competition binding experiment as shown in Fig. 3b, [^3^H]AVP binding to V1b receptors was significantly decreased in the buffer containing 150 mM NaCl or CsCl. At 0 M AVP, the specific binding was reduced to 59 and 53% in the NaCl- and CsCl-containing buffers, respectively. On the other hand, no apparent change in the IC_50_ values was detected. A reduction in the radiolabeled agonist binding by Na^+^ has previously been reported in opioid receptors^22^. The non-competitive mode of inhibition of agonist binding by Na^+^ and Cs^+^ suggests an allosteric effect on V1b receptors.

### Osmolarity is a critical determinant for the internalization of V1b receptors

**Figure 4:**
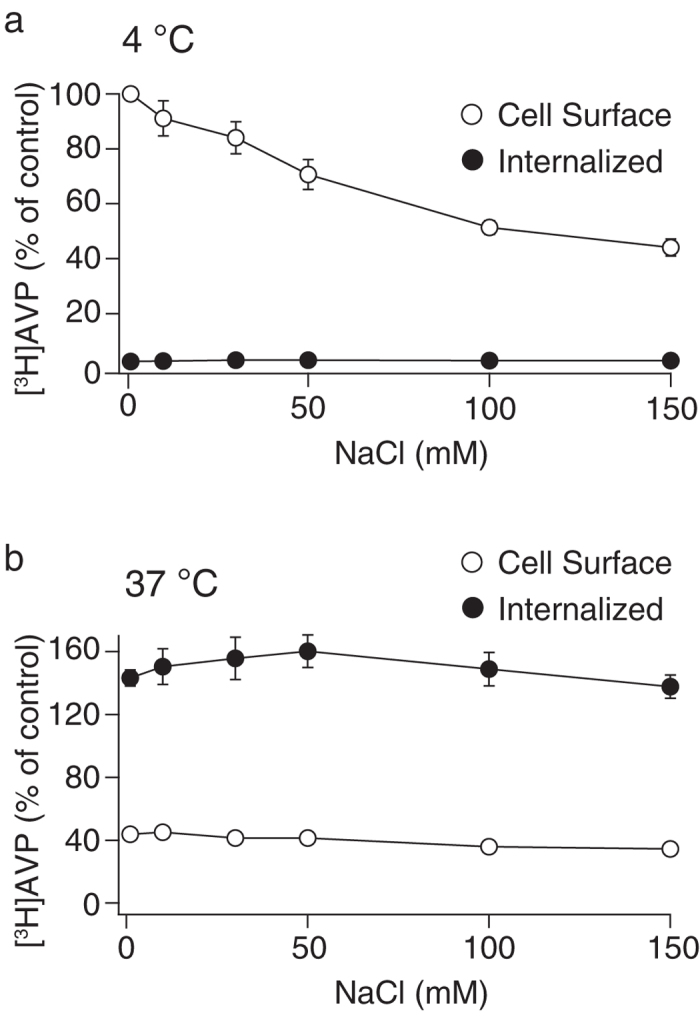
[^3^H]AVP binding and internalization were examined in various NaCl concentrations under constant osmolarity. CHO/V1b cells were used in the binding assay as described in Fig. 1; however, the total osmolarity was maintained constant (300 mOsm) by supplementing with glucose. The results obtained at 4 (**a**) and 37 °C (**b**) incubation are presented. The data are expressed as the percentage of counts obtained from the cells incubated in buffer with no added NaCl at 4 °C, which represents the cell surface counts. Nonspecific binding was determined in the presence of 1 μM AVP. The points in the graph represent the results from four experiments performed in duplicate.

The change in either NaCl or CsCl concentration causes an alteration in the osmolarity of the binding buffer. In the experiments shown in Figs 1 and 2, the change in the osmolarity in the external buffer was not compensated. When glucose was supplemented in the external buffer to maintain osmolarity at a constant value of 300 mOsm, the cell surface [^3^H]AVP binding at 4 °C remained inhibited by the increased concentrations of NaCl (Fig. 4a). However, at 37 °C, the counts from the cell surface and internalized [^3^H]AVP were constant in a wide range of NaCl from 0 to 150 mM, when the osmolarity was maintained constant (Fig. 4b). Therefore, buffer osmolarity is a critical factor for V1b receptor internalization and sensitivity to extracellular NaCl concentrations in a cellular context.

### NH_4_^+^ substituted Na^+^ for the internalization of V1b receptors

**Figure 5:**
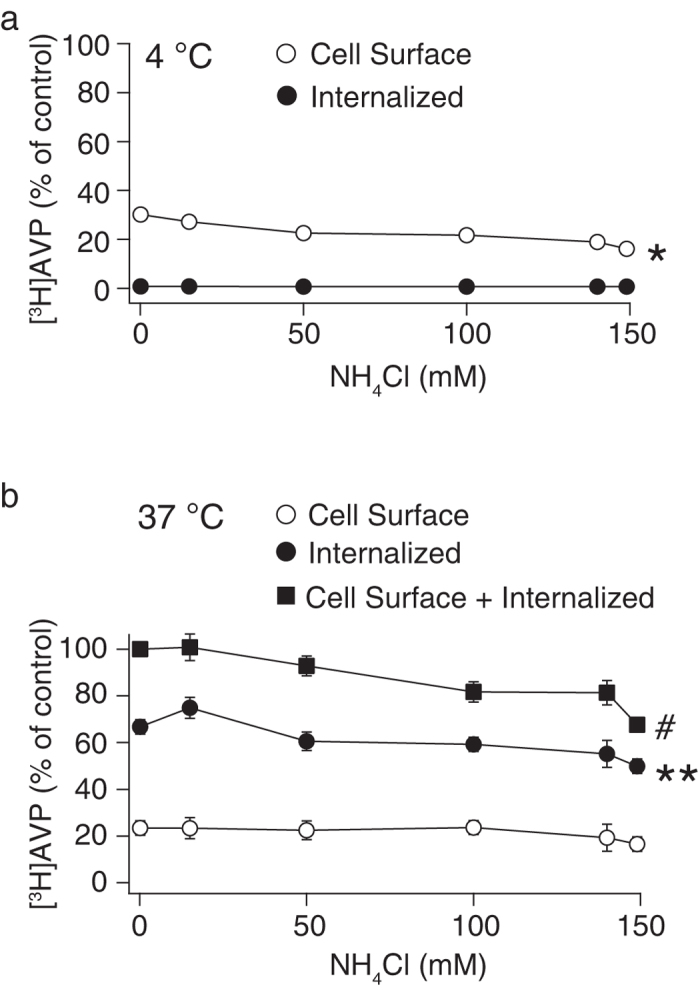
NH_4_^+^ can substitute for Na^+^ in the external buffer to inhibit [^3^H]AVP binding to the V1b receptors. NaCl was gradually substituted with NH_4_Cl, and the pH of the buffer was adjusted to 7.0 ± 0.1. CHO/V1b cells were incubated with 1 nM [^3^H]AVP in each buffer condition at 4 °C for 2 hours to establish the binding equilibrium. The cells were further incubated at 4 (**a**) or 37 °C (**b**) for 30 min and washed with buffer to remove the unbound ligands. Radioactivity associated with the cells was determined by treating the cell with 0.1 N NaOH lysis buffer and mixing the lysis buffer with a scintillation cocktail (filled square in B, *Cell Surface plus Internalized counts*). Washing the cells with acidic washing buffer removed [^3^H]AVP bound to the cell surface receptors. The acidic buffer was recovered and mixed with scintillation cocktail to measure the radioactivity (open circle, *Cell Surface counts*). The cells were lysed after an acid wash, and the remaining counts associated with the cells were measured (filled circle, *Internalized counts*). For the detection of non-specific binding, 1 μM AVP was included in the binding buffer. The data are expressed as the percentage of counts obtained from the cells incubated in 150 mM NaCl and 0 mM NH_4_Cl at 37 °C for 30 min and washed with washing buffer. The data are from four independent experiments. *indicates a significant decrease in the cell surface binding at 150 mM vs. 0 mM NH_4_Cl (p < 0.05). ^#^ and **indicate significant decreases in the cell surface plus internalized binding and internalized binding, respectively (p < 0.05).

When NaCl in the binding buffer was substituted with NH_4_Cl, with the osmolarity of the external buffer maintained at a constant level of 300 mOsm, the inhibition of [^3^H]AVP binding to the cell surface V1b receptors was maintained at all tested concentrations between 0 mM NH_4_Cl (150 mM NaCl) and 150 mM NH_4_Cl (0 mM NaCl; Fig. 5a). Therefore, in contrast to glucose, both Na^+^ and NH_4_^+^ inhibited the [^3^H]AVP binding to the cell surface receptor. By comparing the degree of inhibition between 0 mM NH_4_Cl (150 mM NaCl) and 150 mM NH_4_Cl (0 mM NaCl), we found that the inhibitory effect of NH_4_^+^ on agonist binding was greater than that of Na^+^. (Fig. 5a). At 37 °C, increasing the NH_4_Cl concentration resulted in a small but significant decrease in the internalized [^3^H]AVP; however, the cell surface binding for [^3^H]AVP was not altered (Fig. 5b).

### Effects of anions for the agonist binding and internalization of V1b receptors

**Figure 6:**
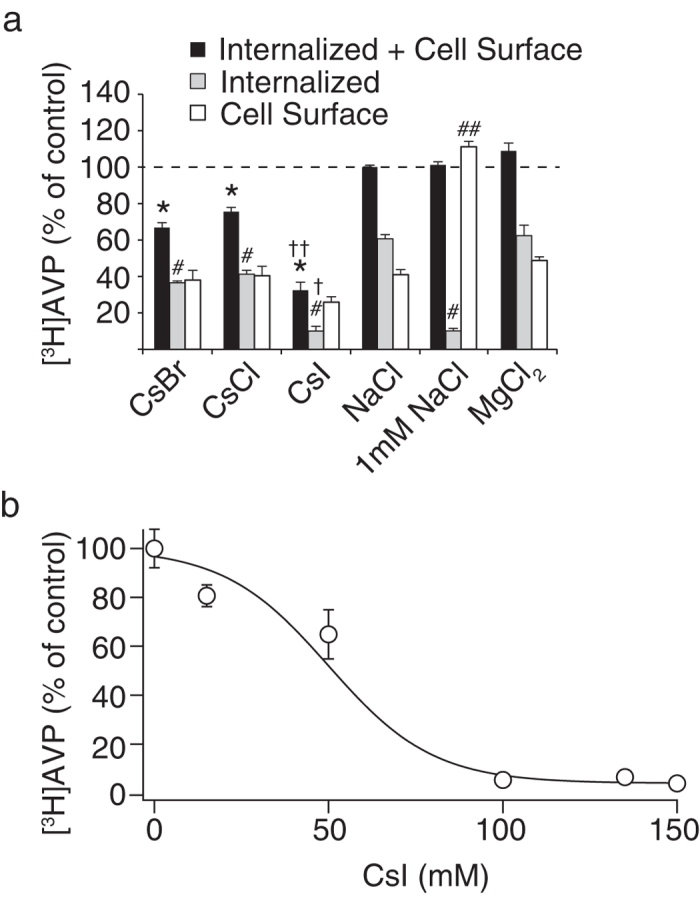
Effects of anion compositions on agonist binding and internalization. (**a**) Using different buffer compositions at 150 mM (CsBr, CsCl, CsI, MgCl_2_, and NaCl) or 1 mM (NaCl), the specific binding of 1 nM [^3^H]AVP to CHO/V1b cells and subsequent internalization were examined. The cells were incubated with 1 nM [^3^H]AVP for 2 hours at 4 °C to establish the binding equilibrium and were further incubated at 37 °C for 30 min to induce receptor internalization. The cells were washed with binding buffer at 4 °C to remove excess radioligand, and collected to measure the *Internalized plus Cell Surface* counts (black bars). The *Internalized* counts (gray bars) and *Cell Surface* counts (white bars) were measured as described in the Materials and methods. *indicates a significant decrease in the total counts (150 mM NaCl vs. CsBr, CsCl, or CsI, p < 0.05). ^#^indicates significant decrease in *Internalized* counts (150 mM NaCl vs. CsBr, CsCl, or CsI, or 1 mM NaCl, p < 0.05). ^##^indicates a significant increase in *Cell Surface* counts (150 mM NaCl vs. 1 mM NaCl). ^†^ and ^††^indicate significant decreases in the *Internalized plus Cell Surface* and *Internalized* counts, respectively, in CsI or CsBr buffer compared to the CsCl buffer. The data are from four to seven independent experiments. (**b**) I^−^ ion inhibited [^3^H]AVP binding to the V1b receptors. Membrane samples (70 μg) from CHO/V1b cells were incubated with 1 nM [^3^H]AVP in 400 μL of binding buffer with the different concentrations of CsI. CsCl was supplemented to maintain osmolarity at constant value of 300 mM. Nonspecific binding was determined in the presence of 1 μM AVP. The points in the graph represent results from four experiments performed in duplicate.

We further investigated whether different anions in the external buffer have an influence on agonist binding and receptor internalization, although the anion binding site has not been defined in structural studies of GPCRs. To examine the effects of changing the anion composition, we implemented cesium salts, instead of sodium salts, to exclude the potent promoting effect of Na^+^ on the agonist-induced internalization. The specific counts of [^3^H]AVP associated with the CHO/V1b cells, which were composed of cell surface and internalized agonists, were smaller in 150 mM CsBr, CsCl or CsI compared with those in 150 mM NaCl (Fig. 6a, black bars), which was mainly because of decreases in the internalized counts in the CsBr, CsCl or CsI buffer compared with the NaCl buffer (Fig. 6a, gray bars). The amount of [^3^H]AVP associated with the cell surface receptors was not significantly altered (Fig. 6a, white bars). Furthermore, the acid resistant, internalized counts were further decreased in the buffer containing 150 mM CsI compared with the CsCl or CsBr buffer (Fig. 6a, gray bars). Therefore, internalization of the V1b receptors was dependent on not only the Na^+^ levels but also the I^−^ levels in the external buffer. Substitution of Na^+^ to Mg^2+^ did not change the agonist binding or agonist-mediated internalization of the V1b receptors (Fig. 6a). In the experiment using radioligand binding to membrane preparations, changing the buffer composition from CsCl to CsI while maintaining a constant overall cesium salt concentration of 150 mM, resulted in a decrease in the [^3^H]AVP binding (Fig. 6b). At 150 mM CsI, the specific [^3^H]AVP binding to membrane preparations from CHO/V1b cells decreased to 4% of the binding detected in 150 mM CsCl (n = 3). The IC_50_ value of CsI was 50 ± 2 mM. These findings indicated that anion species in the buffer are the critical determinants of [^3^H]AVP binding to the V1b receptors.

### Effects of KCl on the agonist binding and internalization of V1b receptors

**Figure 7:**
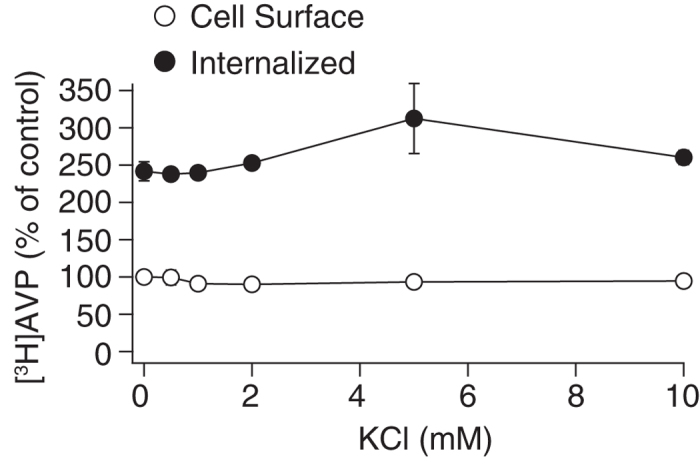
Effects of reducing KCl in assay buffer on the agonist binding and internalization of V1b receptors. KCl was reduced from 10 to 0 mM without compensation of osmolarity. CHO/V1b cells were incubated with 1 nM [^3^H]AVP in buffer with different KCl concentrations at 4 °C for 2 hours to establish the binding equilibrium. The cells were further incubated at 37 °C for 30 min to induce internalization. The cells were subsequently washed with neutral buffer to remove the unbound ligands. Washing the cells with an acidic buffer removed [^3^H]AVP bound to the cell surface receptor, and the acidic buffer was mixed with a scintillation cocktail to measure radioactivity (open circle). The cells were lysed after an acid wash, and the remaining counts associated with the cells were measured (filled circle). For the detection of non-specific binding, 1 μM AVP was included in the binding buffer. The data are expressed as the percentage of cell surface counts incubated with no added KCl. The points in the graph represent the results from four experiments performed in duplicate.

Finally, we examined the effect of reducing KCl in the external buffer on the agonist binding and internalization of V1b receptors. As shown in Fig. 7, a decrease in the external K^+^ levels had no apparent effect on the agonist counts; however, the cellular localization of the V1b receptor (cell surface or cytoplasm) had a significant effect in a two-way ANOVA analysis (P = 0.7 for KCl concentration; P < 0.01 for cellular localization of V1b receptors, surface/intracellular; no interaction was identified between the KCl concentration and cellular localization). Therefore, in our experimental conditions, extracellular Na^+^ has a more profound effect on V1b receptor internalization compared with K^+^.

## Discussion

We searched for ionic and osmolar conditions in the external buffer that enabled efficient agonist binding to GPCRs and reduced receptor internalization. V1b receptors in CHO cells serve as an ideal model for investigating this topic, because in the unstimulated state, only a small fraction of the V1b receptor is located in the plasma membrane, and a substantial fraction of the receptor is in the cytoplasm. In addition, the cell surface V1b receptors extensively internalize upon agonist binding. Therefore, it is difficult to characterize V1b receptors in a cellular context. At a physiological concentration of 135 mM NaCl in the external buffer (Fig. 1b), the fraction of internalized agonist reached 106% of the initial cell surface receptor, suggesting that the V1b receptor that initially resided in the intracellular space was inserted to the plasma membrane and further internalized following agonist binding^20^. This extensive internalization of the V1b receptors was maintained in a wide range of external NaCl concentrations (50–150 mM). At NaCl concentrations of less than 50 mM, agonist binding to the cell surface V1b receptors was sharply increased with the reduction of the Na^+^-mediated binding blocking (Fig. 1b). Therefore, the change in external Na^+^ concentration had a significant impact on the amount and kinetics of agonist binding to the cell surface V1b receptors. One interesting and new finding in the current study is the preference of Na^+^ to Cs^+^ in the external buffer preceding V1b receptor internalization. In addition to ionic compositions, osmolarity is a critical determinant for V1b receptor internalization. Furthermore, we found that I^−^ inhibited agonist binding to the V1b receptors.

[^3^H]AVP binding to the cell surface V1b receptor was inhibited by increasing the Na^+^ level in the external buffer. The aspartic acid in the second transmembrane domain, Asp^2.50^, plays a critical role in Na^+^-mediated blocking of agonist binding to many class A GPCRs^3^. This aspartic acid is conserved among all members of the vasopressin/oxytocin receptor family including the V1b receptors. It has been reported that the extent of Na^+^-dependent inhibition of agonist binding differs depending on the receptor examined. In human histamine receptors, the activity of the H3-receptor (hH3R) is sensitive to Na^+^; however, the constitutive and agonist-dependent activity of hH4R is not^23^. Agonist binding to the Y_1_ subtype neuropeptide Y receptor is selectively enabled by Ca^2+^ and strongly attenuated by Na^+^ and other alkali monovalent cations, whereas monovalent cations including Na^+^ up to 100 mM stimulate the agonist binding to Y_2_ receptors, and inhibit binding at higher concentrations^24^,^25^. Therefore, it is important to experimentally define the Na^+^ effect in each receptor.

A reduction in the NaCl concentration below 50 mM in the binding buffer without compensation of osmolarity decreased the V1b receptor internalization. Maintaining the osmolarity at a physiological level by supplying glucose or NH_4_Cl in a low-NaCl buffer caused the recovery of receptor internalization. However, glucose did not substitute for the role of NaCl in terms of the Na^+^-mediated inhibition of the agonist binding. Extensive internalization of V1b receptors and accumulation of radiolabeled agonist in the cells enabled the internalization assay of V1b receptors in good signal-to-noise ratio. On the other hand, the internalization of V1a receptors is less extensive compared with V1b receptors from our previous analysis^20^. Moreover, internalized V1a receptors rapidly recycle to the plasma membrane^26^. V2 receptors internalize for a longer period than V1a receptors; however, internalized fraction was approximately 30% of initial cell surface count^26^. We demonstrated that extensive internalization of V1b receptors is based on the sensitivity to Na^+^ ions. The robustness of the V1b receptor internalization was observed when external buffer contained reduced concentrations of Na^+^ but not Cs^+^. In previous reports, hypotonic shock and the subsequent treatment of a cell with low-potassium buffer has been demonstrated to inhibit internalization of LDL- or EGF-bound receptors^8^. Intracellular potassium is necessary for formation of new coated pits, whereas already assembled coated pits can internalize receptor-bound LDL^9^. In the protocol for the sequential treatment of hypotonic shock and low-potassium buffer, the osmolarity of the hypotonic buffer was reduced to half of the physiological culture medium^9^. We demonstrated that the hypotonic condition, which was produced by reducing Na^+^ concentration, was sufficient to inhibit V1b receptor internalization and the treatment of cells in the low-potassium buffer was not necessary. Furthermore, decreasing NaCl concentration in the external environment decreased the internalization of the V1b receptors, despite the increased agonist binding to the cell surface receptor. The increase in the cell surface receptor without internalization by lowering external medium osmolarity (<50 mM NaCl) may have practical implications for efficient drug screening of hydrophilic candidates with agonist activity.

We demonstrated that extracellular Na^+^ is more effective than Cs^+^ in promotion of receptor internalization (Fig. 2c). Many reports have described chemical and genetic tools that control clathrin-mediated internalization^27^. To the best of our knowledge, this investigation is the first study that demonstrates clear preference for Na^+^ compared with Cs^+^ in agonist-mediated internalization of a member of the GPCR family. Na^+^-mediated promotion of the internalization of the V1b receptor was not a result of the increase of agonist binding to the V1b receptors; our experiments on [^3^H]AVP binding using membrane preparation and whole cells revealed that inhibitory effects on the agonist binding were comparable between NaCl- and CsCl-containing buffers. In contrast, previous work has demonstrated that the influence of Na^+^ on opiate receptor binding is highly specific^22^. The inhibition of opioid agonist binding by Na^+^ was mimicked only with Li^+^, but not with alkali ions with larger ionic radii^22^. We selected CsCl to compare with NaCl, because the ionic radius of Cs^+^ is reportedly the largest among stable alkali monovalent cations^28^. We found that the Na^+^ binding site in the V1b receptor does not recognize the difference between Na^+^ and Cs^+^, in contrast to the mechanism for Na^+^-mediated promotion of internalization. The molecular identity of the preference for Na^+^ over Cs^+^ is currently speculative. A structural study of the inactive and active-like states of A_2A_ adenosine receptors has suggested that the Na^+^ binding pocket is collapsed in agonist-bound receptors. This suggests that agonist-bound receptors, which proceed to internalization, may not be relevant for Na^+^ sensitivity during the internalization process.

Hypotonicity changed the cellular distribution of the V1b receptors in the plasma membrane and the cytoplasm. In a previous report, osmotic cell-swelling in the hypotonic environment has been shown to increase cell surface area in intestine 407 cells, as well as increase their subsequent uptake of tetramethylrhodamine isothiocyanate (TRITC)-dextran^29^. The preceding increase in cell surface area is due to an increase in exocytosis regulated by ATP that was released to extracellular space by hypotonic shock^29^,^30^,^31^. Extracellular ATP can act in both an autocrine and paracrine manner to stimulate purinergic receptors, P_2Y_ GPCRs or P_2X_ ligand-gated ion channels, leading to an increase in the phosphorylated form of ERKs^29^,^32^. Subtypes of the P_2Y_ receptor are known to couple with G_q/11_ proteins, as do the V1b receptors. It is tempting to speculate that the G_q/11_-coupled V1b receptors might use the same signaling cascade as ATP to regulate receptor internalization and recycling. Future studies should elucidate whether ATP could add further regulation to the cellular trafficking of the agonist-stimulated V1b receptors.

Monovalent anions were found to have a significant inhibitory impact on AVP binding to the V1b receptors. We selected cesium salts to examine a possible role of anions in agonist binding and internalization because Na^+^ significantly promotes receptor internalization and may obscure differences among anions. Our analysis revealed that I^−^, but not Br^−^, reduced [^3^H]AVP internalization. Moreover, increasing the concentration ratio of CsI/CsCl in the binding buffer while keeping the total cesium salts at 150 mM resulted in the inhibition of specific [^3^H]AVP binding. Therefore, we concluded that I^−^ has a large inhibitory impact on the binding of AVP to V1b receptors and the subsequent internalization.

The role of I^−^ in receptor-effector coupling has been reported in previous studies. Agonist stimulation of the β_2_-adrenergic receptor enhances adenylate cyclase activity more efficiently in NaI buffer compared with NaCl buffer^33^. There is a strong linear correlation between the anion radius and the efficiency of sodium salts for enhancing the adenylate cyclase^33^. For CXCR_4_ chemokine receptors, maximum response and agonist potency in GTP hydrolysis are decreased more in NaI buffer than in NaCl buffer^34^. Iodide ions may increase the affinity of GDP to interact with Gi protein, which results in inefficient GDT-GTP exchange^34^. Furthermore, NaI inhibits signaling via H3 histamine receptors and binding of GPTγS to δ-opioid-G_i_ fusion receptors^35^,^36^. We identified strong inhibitory effects of I^−^ on agonist binding to the V1b receptor. It has been known that coupling between the receptor and GTP-bound form of G_α_-proteins usually decreases the affinity of the agonist^37^. Further investigation is necessary to clarify whether I^−^ inhibits the GTPase activity of G_α_-proteins coupled with the V1b receptors and increases the GTP-bound form of G_α_-proteins. Although the mechanism of I–mediated inhibition of agonist binding to the V1b receptor is not clear at present, the use of buffer containing Cs salts was effective to elucidate the effects of each anion on the agonist binding to V1b receptors.

Our findings indicate that the buffer compositions for optimal V1b receptor binding and internalization are different from the buffer compositions for promoting transferrin internalization in reticulocytes^38^. Approximately 50–80 mM NaCl in the buffer is necessary to reach a submaximal internalization of both iron-bound transferrin and AVP bound to the V1b receptors^38^. However, NH_4_Cl cannot substitute for NaCl in the transferrin internalization; the level of iron internalization does not increase with a rise in NH_4_Cl concentration in NaCl-reduced buffer^38^. In V1b-mediated internalization, 50% of the total counts associated with the cells were from the internalized ligand in 150 mM NH_4_Cl compared with 67% in 150 mM NaCl (Fig. 5). Together with the result that NH_4_^+^ can be substituted with Na^+^ for inhibition of AVP binding, the role of NH_4_^+^ in V1b receptor-mediated internalization is different from the role in transferrin-mediated internalization. Additionally, sucrose cannot be substituted with NaCl in transferrin internalization in contrast to V1b receptor internalization^38^. Therefore, a different external environment appears to be necessary for the internalization of the V1b receptor in the GPCR family compared with the internalization of the transferrin receptor of the single-transmembrane receptor family^39^.

In summary, external ionic conditions have substantial impacts on agonist binding and V1b receptor internalization, which have not been previously recognized. A reduction in the NaCl concentrations increased cell surface binding mainly because of the following two reasons: an increase in the agonist affinity and a decrease in the receptor internalization in CHO/V1b cells. The mechanism of Na^+^-sensitive receptor internalization should be further investigated.

## Methods

### Materials

Construction of the expression plasmid containing a coding sequence of the mouse V1b receptor was described previously^40^. Chinese hamster ovary (CHO)-derived cells were obtained from the American Type Culture Collection (Rockville, MD, USA). Reagent grade chemicals were purchased from Wako Pure Chemical Industries (Osaka, Japan). [^3^H]AVP (61.2 Ci/mmol) was purchased from PerkinElmer Japan (Yokohama, Japan).

### Cell culture and transfection

CHO cells were cultured in Ham’s F12 medium supplemented with heat-inactivated 10% fetal calf serum (Life Technologies Japan, Tokyo, Japan), 100 U/mL penicillin, and 100 g/mL streptomycin at 37 °C in 5% CO_2_ in an air-ventilated humidified incubator. Production of CHO cell lines that stably express V1b receptors (CHO/V1b) was described previously^20^,^41^. Briefly, one day after seeding the 1 × 10^5^ cells in a 100-mm cell culture dish, the medium was supplemented with a mixture of 0.7 mL of serum-free F12 medium, 21 μL of FuGene 6 transfection reagent (Promega, Tokyo, Japan) and 7 μg of the plasmid DNA, and the cells were cultured for 24 hours. The cells were collected with 0.025% trypsin and 0.01% EDTA treatment and seeded at 1 × 10^4^ cells in 100-mm tissue culture dishes. Single colonies that were resistant to the treatment of 0.2 mg/mL zeocin (Thermo Fisher Scientific, Waltham, MA, USA) were isolated and maintained in the same selection medium. The expression of the transfected receptors was screened using intracellular Ca^2+^ measurement^42^. We previously reported the expression levels and agonist affinity of the V1b receptors in this cell line (Bmax = 645 ± 53 fmol/mg protein and Kd = 1.8 ± 0.7 nM)^20^.

### Radioligand binding

In 12-well dishes, cells were plated at a density of 1 × 10^5^ cells/well, incubated for 24 hours and used for a radioligand binding assay^20^,^41^. For the saturation binding studies, the cells were washed once with ice-cold Ham’s F-12 medium and incubated for 2 hours on ice with 10–10000 pM of [^3^H]AVP in buffer C [Ham’s F-12 medium containing 50 mM HEPES (pH = 7.4), 0.3% bovine serum albumin]. The cells were then washed 3 times with buffer B (50 mM Tris-HCl, pH = 7.4, 10 mM MgCl_2_, and 0.3% bovine serum albumin), collected with 0.5 mL of 0.1 N NaOH, and mixed with 4 mL of the scintillation cocktail (ULTIMA Gold^TM^, PerkinElmer, MA, USA). The total binding sites and Kd values were calculated using the nonlinear curve-fitting computer program Igor (WaveMetrics, Lake Oswego, OR, USA). There was no detectable specific binding or uptake of [^3^H]AVP for the wild-type CHO cells (see Supplemental Figure SF1).

To measure receptor internalization, cells in 12-well culture plates were treated with 1 nM [^3^H]AVP in assay buffer [10 mM HEPES (pH = 7.4), 5 mM KCl, 1.2 mM CaCl_2_, 1 mM MgCl_2_, and 10 mM glucose] containing various concentrations of NaCl and/or glucose for 2 hours at 4 °C. The culture plates were subsequently incubated at 37 or 4 °C for 30 min. Following the incubations, all plates were washed three times with ice-cold neutral buffer at 4 °C to remove excess [^3^H]AVP. [^3^H]AVP bound to cell surface receptors was removed by acid washing with ice-cold acidic buffer (50 mM sodium acetate and 150 mM NaCl, pH = 3) for 5 min at 4 °C^43^. The remaining counts following the acid wash were considered to come from internalized [^3^H]AVP.

Membrane samples for the radioligand binding study were prepared from culture cells as previously described^44^. For the saturation binding studies, 25 μg of the membrane samples were incubated with 50–10000 pM [^3^H]AVP for 60 min at 25 °C in 400 μL of binding buffer B. At the end of the incubation period, 1 mL of ice-cold buffer B was added, and the samples were filtered through a glass fiber membrane (type GF/B, GE Healthcare UK Ltd., UK). After three washes with buffer B, the filters were soaked in 4 mL of liquid scintillation cocktail and the membrane-bound radioactivity was measured. The nonspecific radioligand binding was determined in the presence of 1 μM AVP.

### Data analysis

All values in the text are reported as the mean ± SEM. In several data points in the Figures, the errors are so small that the error bars are behind the symbol. Significant differences were determined by Student’s t-test or ANOVA followed by a multiple comparison test with Holm’s adjustment. The statistics were calculated using the statistical computer program R [R Core Team (2015). R: A language and environment for statistical computing. R Foundation for Statistical Computing, Vienna, Austria].

## Additional Information

**How to cite this article**: Koshimizu, T.-a. *et al.* Combined sodium ion sensitivity in agonist binding and internalization of vasopressin V1b receptors. *Sci. Rep.* **6**, 25327; doi: 10.1038/srep25327 (2016).

## Supplementary Material

Supplementary Information
