# Supplementary material for: Combined sodium ion sensitivity in agonist binding and internalization of vasopressin V1b receptors
Source: Sci Rep. 2016 May 3;6:25327. doi: 10.1038/srep25327 (PMC4853784; doi:10.1038/srep25327)
Supplement: Supplementary Information [file srep25327-s1.pdf]

Supplementary Information

Combined sodium ion sensitivity in agonist binding and internalization of vasopressin V1b receptors

Taka-aki Koshimizu[1,*], Aki Kashiwazaki[1], and Junichi Taniguchi[1]

[1]Division of Molecular Pharmacology, Department of Pharmacology, Jichi Medical University, Tochigi 329-0498, Japan

*, corresponding author t koshi@jichi.ac.jp

Supplementary Figure SF1

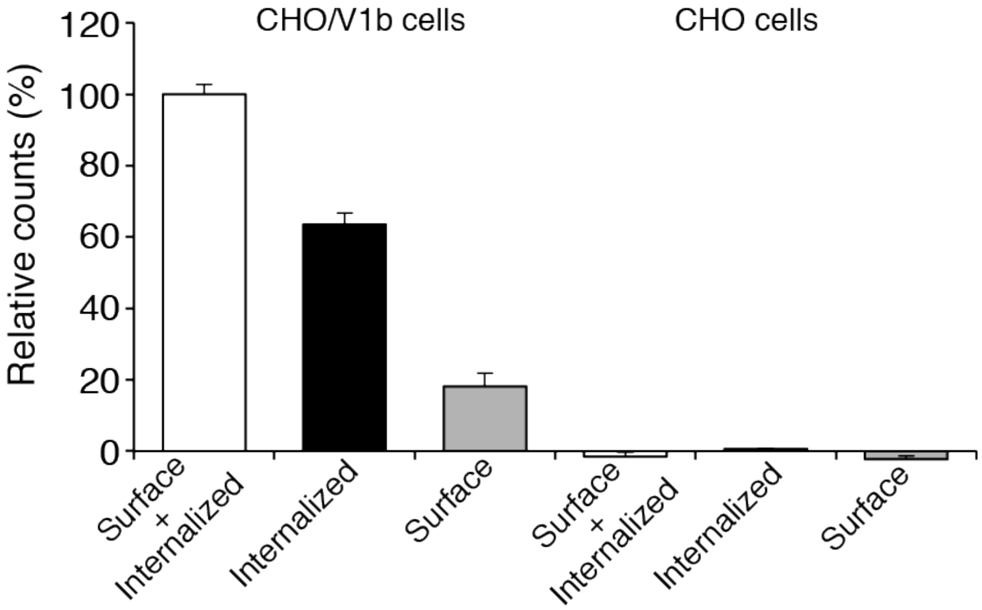

Figure legend for Supplementary Figure S1

The specific binding site for tritium-labeled arginine vasopressin ([^{3}H]AVP) cannot be detected in CHO cells. Untransfected Chinese hamster ovary (CHO) cells and CHO cells that stably express V1b receptors (CHO/V1b cells) were plated on 12-well dishes at 10^5 cell/well. On the next day, the cells were washed once with Assay buffer [10 mM

HEPES (pH = 7.4), 135 mM NaCl, 5 mM KCl, 1.2 mM $CaCl_2$, 1 mM $MgCl_2$, 10 mM glucose, and 0.3% bovine serum albumin] and incubated in 450 μL of 1 nM [^{3}H]AVP in the Assay buffer at 4 °C for 2 hours. Internalization was initiated by transferring the dishes to an air incubator at 37 °C and incubating for 30 min in this condition. The cells were washed three times with ice-cold Washing buffer (50 mM Tris-HCl, pH = 7.4, 10 mM $MgCl_2$) and collected with 500 μL of 0.1 N NaOH for the total counts (Surface+Internalized). [^{3}H]AVP bound to the cell-surface receptors was recovered via incubation of the cells in 500 μL of acidic buffer (50 mM sodium acetate, 150 mM NaCl, pH = 3) on ice for 10 min (Surface). After washing with acidic buffer, the remaining cells were collected with 500 μL of 0.1 N NaOH for the internalized counts (Internalized). Specific binding sites for [^{3}H]AVP were not identified in the untransfected CHO cells. Counts were normalized by the Surface+Internalized counts, which were identified in the CHO/V1b cells. Non-specific binding was determined in the presence of 1 μM AVP. The data are from three independent experiments performed in triplicate and are expressed as the mean ± S.E.M.
